# Topical steroid and non-steroidal anti-inflammatory drugs inhibit inflammatory cytokine expression on the ocular surface in the botulium toxin B-induced murine dry eye model

**Published:** 2012-07-03

**Authors:** Lei Zhu, Cheng Zhang, Roy S. Chuck

**Affiliations:** 1 Henan Eye Institute, Zhengzhou, China; 2 Department of Ophthalmology and Visual Sciences, Albert Einstein College of Medicine, Montefiore Medical Center, Bronx, NY

## Abstract

**Purpose:**

To evaluate the effect of the topical steroid, fluorometholone, and the non-steroidal anti-inflammatory drugs (NSAIDs), nepafenac and ketorolac, on inflammatory cytokine expression of the ocular surface in the botulium toxin B-induced murine dry eye model.

**Methods:**

Topical artificial tears (0.5% carboxymethylcellulose sodium), 0.1% fluorometholone, 0.1% nepafenac, and 0.4% ketorolac were applied 3 times per day in a dry eye mouse model 1 week after intralacrimal botulium toxin B (BTX-B) or saline (sham) injection. Tear production and corneal fluorescein staining were evaluated in all groups before injection at baseline and at 3 time points up to 4 weeks after injection. The pro-inflammatory cytokines interleukin-1β (IL-1β) and tumor necrosis factor-α (TNF-α) were evaluated by immunohistochemistry.

**Results:**

BTX-B-injected mice showed significantly decreased aqueous tear production and increased corneal fluorescein staining at the 1 and 2 week time points compared with normal control and saline-injected mice. In the BTX-B-injected mice, immunoﬂuorescent staining for TNF-α and IL-1β in corneal and conjunctival epithelial cells increased significantly at the 2 and 4 week time points compared to that of normal and saline-injected mice, and returned to normal levels at the 4 week time point. Topical fluorometholone significantly improved corneal surface staining in the BTX-B-injected mice after 1 week of treatment, and increased the tear production within 2 weeks, but without statistical significant difference. Topical fluorometholone significantly decreased the staining of TNF-α and IL-1β in corneal and conjunctival epithelia after 1-week treatment. Topical artificial tears, 0.1% nepafenac, and 0.4% ketorolac did not show obvious effects on tear production, corneal surface staining, and levels of IL-1β and TNF-α expression in normal, and BTX-B-injected dry eye mice.

**Conclusions:**

Topical fluorometholone caused suppression of inflammatory cytokine expression on the ocular surface in the Botulium toxin B-induced murine dry eye model, while topical NSAIDs demonstrated no clearly beneficial effects.

## Introduction

Dry eye disease is a very common disorder which refers to a spectrum of ocular surface diseases with multiple etiologies [1]. Regardless of the initiating causes, a vicious cycle of inflammation can develop on the ocular surface in dry eye that leads to ocular surface disease [2]. Anti-inflammatory therapy may be beneficial for dry eye treatment which has been reported in a series of animal and clinical trials. Anti-inflammatory agents have become one of the mainstays of therapy for dry eye syndrome [3-6].

The botulium toxin B (BTX-B)-induced mouse tear-deficiency dry eye model has been developed and shown to mimic human non-Sjögren's disease. Lacrimal gland injection of BTX-B resulted in ocular surface changes such as corneal fluorescein staining and significantly decreased tear production without detectable inflammation of the lacrimal glands [7,8]. Our recent study demonstrated that ocular surface inflammation develops in the BTX-B-induced dry eye mouse model [9]. Interleukin-1β (*IL-1β*) and tumor necrosis factor-α (*TNF-α*) mRNA expression in the corneal and conjunctival epithelia significantly increased in BTX-B-injected mice at 1 and 2 weeks after injection, and returned to baseline at 4 weeks. Immunohistochemical study of these cytokines supports the findings of increased gene expression in this animal model [9]. These changes were also consistent with observed alterations in tear production and on the ocular surface in other dry eye animal models as well [9].

This current study was designed to investigate whether topical fluorometholone, ketorolac and nepafenac inhibit inflammatory cytokine expression on the ocular surface in the BTX-B-induced murine dry eye model.

## Methods

### Animal model

Female 6–8 week-old CBA/J mice (Jackson Laboratories, Bar Harber, ME) were used in this study in accordance with the ARVO Statement for the Use of Animal in Ophthalmic and Vision Research.

Mice were divided into 3 groups, including a normal control group without any lacrimal injection and treatment, a saline-injected sham group and a BTX-B-injected group. One week after intra-lacrimal gland injection, the saline-injected sham group and BTX-B-injected groups were randomized into 4 subgroups separately (9 mice in each group) to receive topical treatments with 0.5% carboxymethylcellulose sodium, 0.1% fluorometholone (Allergan, Inc. Irvine, CA), 0.1% nepafenac (Alcon Laboratories, Inc. Fort Worth, TX), or 0.4% ketorolac (Allergan, Inc. Irvine, CA). One microliter of each eye drop was applied 3 times per day. The mouse model was created using a previously reported method [7]. Briefly, all mice in saline-injected sham group and BTX-B-injected group were anesthetized with Ketamine and Xylazine (45 mg/kg and 4.5 mg/kg, respectively). Saline (0.05 ml) or BTX-B (0.05 ml, 20mU. Myobloc™; Elan Pharmaceuticals Inc., South San Francisco, CA) was injected into the right lacrimal gland unilaterally through the conjunctiva with custom made 33-gauge needle (Hamilton, Reno, NV) under an operating microscope. All mice were maintained under relatively constant temperature (21 °C to 24 °C) and humidity conditions (<20%).

### Measurement of aqueous tear production and corneal fluorescein staining

Measurements of aqueous tear production and corneal fluorescein staining were performed as previously reported [7]. Tear volume was measured with phenol red-impregnated cotton threads (Zone-Quick; Oasis, Glendora, CA). The cotton threads were applied to the ocular surface in the lateral canthus for 15 s in the non-anesthetized mouse, and the wet red threads were measured in millimeters. Tear volume was measured in all mice before lacrimal gland injection and 1, 2, and 4 weeks after injection. Corneal fluorescein staining (1μl of 1% sodium fluorescein; Sigma-Aldrich, St. Louis, MO) was evaluated under cobalt blue light with a grading system based on area of corneal staining as previously reported [7]. The area of punctate staining was designated as grade 0 when there was no punctate staining, grade 1 when equal to or less than one eighth was stained, grade 2 when equal to or less than one fourth was stained, grade 3 when equal to or less than one half was stained, and grade 4 when greater than one half or the entire area was stained. All measurements were performed before injection and at 1, 2, and 4 weeks after injection.

### Immunohistochemistry

Based on previous studies, inflammatory cytokines TNF-α and IL-1β were chosen for immunofluorescent staining [8,9]. The mice from each group (normal control group, BTX-B and saline sham lacrimal gland injection) were sacrificed at 4 time points (before injection, and 1, 2, and 4 weeks after injection). The cornea and conjunctiva from each group at different time points were harvested by dissection and immersed in 4% paraformaldehyde, fixed overnight at 4 °C, then embedded in optimal cutting temperature embedding compound (OCT; Tissue-Tek®; Sakura, Terrence, CA). Sagittal frozen sections were cut at 10 μm thickness. After drying at room temperature for 15 min, slides were washed twice with phosphate buffered saline (PBS) and incubated with blocking serum for 30 min, then incubated with primary antibodies overnight at 4 °C. The antibodies used were as follows: anti-IL-1β (rabbit polyclonal IgG; 1:400; Santa Cruz Biotechnology, Inc., Santa Cruz, CA), anti-TNF-α (goat polyclonal IgG; 1:200; Santa Cruz Biotechnology). Slides were washed with PBS, then secondary antibodies conjugated with either Cy3 or Cy2 (1:200; Jackson ImmunoResearch, West Grove, PA) were applied and incubated for 1 h at room temperature. After washing with PBS, slides were counterstained with Hoechst 33342 nuclear staining dye (1:4,000; Molecular Probes, Eugene, OR) for 30 s and mounted with Mounting Media (Dako, Carpinteria, CA). Negative control experiments were performed by omitting the primary antibody. Isotype controls were performed by using normal rabbit IgG (1:400) or normal goat IgG (1:100; Santa Cruz Biotechnology). Sections were visualized with a digital fluorescence microscope (Eclipse; E1000; Nikon, Tokyo, Japan). Images for each slide were obtained with the same settings. Quantification of immunofluorescence was performed (Nikon Eclipse 80i, NIS-Elements AR3.1).

### Statistical analysis

Statistical analyses were performed using SPSS for Windows version 10.0 and the two way *t*-test. Two-way ANOVA was used for the multiple comparisons between different time points. Correlations between the intensity of fluorescence and corneal fluorescein staining or tear production were performed. P-values of less than 0.05 were considered statistically significant.

## Results

### Aqueous tear production and corneal staining

**Figure 1:**
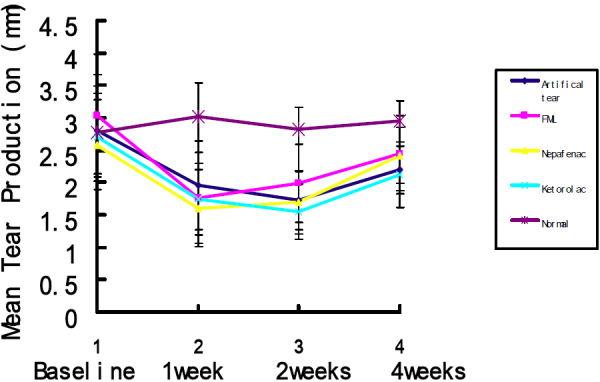
Tear production in different groups with BTX-B injection at different time points after injection. The reduction of aqueous tear production persisted during the observation period in all groups. The tear production of fluorometholone (FML)-treated group tended to increase within 2 weeks, but was not statistically different. (Compared to baseline, the tear production is still significantly lower, p=0.035).

All mice retained full blink function after BTX-B injection. BTX-B-injected mice showed significantly decreased aqueous tear production at 1-week (1.95±0.69 mm [mean±SD]) and 2-week (1.73±0.45 mm) time points compared with normal control (3.02±0.0.52 mm) and saline-injected mice (1 week 2.86±0.55 mm and 2 weeks 3.14±0.86 mm post-injection; p<0.01). The reduction of aqueous tear production persisted during the majority of the observation period, tended to increase at the 4-week time point (2.19±0.36 mm), but was not statistically significant compared to normal control and saline-injected mice (p=0.126, p=0.077; respectively). Topical fluorometholone-treated mice showed increased tear production at the 1 week time point, but still lower than normal and saline-injected mice significantly (p=0.027, p=0.0418, respectively). Topical artificial tears, 0.1% nepafenac, and 0.4% ketorolac did not show obvious effects on tear production. Tear production in all groups of mice returned to normal at 4 weeks (Figure 1).

**Figure 2:**
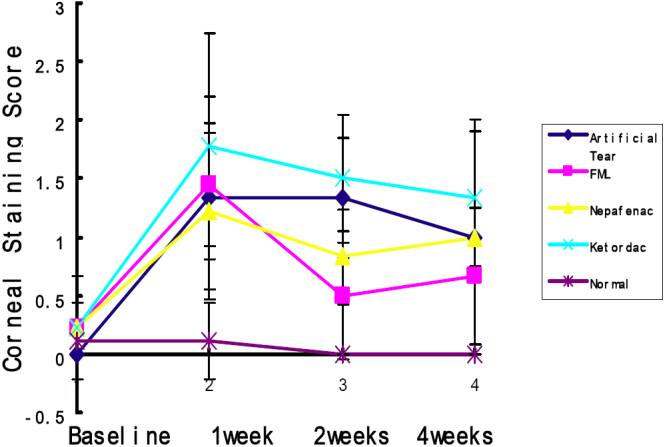
Corneal fluorescein staining scores in all groups with BTX-B injection at different time points after injection. Average score in all groups was significantly increased after BTX-B injection during the observation period, but only FML-treated groups returned to normal within 2 weeks. (Compared to baseline, p=0.148).

There were no significant differences in corneal fluorescein staining score between the groups at baseline. Little or no corneal staining in saline-injected and normal control mice was observed at the various time points after injection. BTX-B-injected mice showed significantly increased corneal fluorescein staining at 1-week time point in all groups. Topical fluorometholone-treated mice demonstrated significant improvement in corneal fluorescein staining at the 2-week and 4-week time points, while in other treatment groups corneal fluorescein staining seemed to improve, but without statistical significance during the observation period (Figure 2). Spearman correlation analysis showed significant positive correlation between the intensity of fluorescence (TNF-α, IL-1β) and corneal fluorescein staining in the FML-treated group (correlation coefficients: 0.492 and 0.559, p=0.045 and 0.024, respectively), but other groups exhibited no association. The intensity of fluorescence (TNF-α, IL-1β) showed no linear association with tear production in any groups.

### Immunohistochemistry

**Figure 3:**
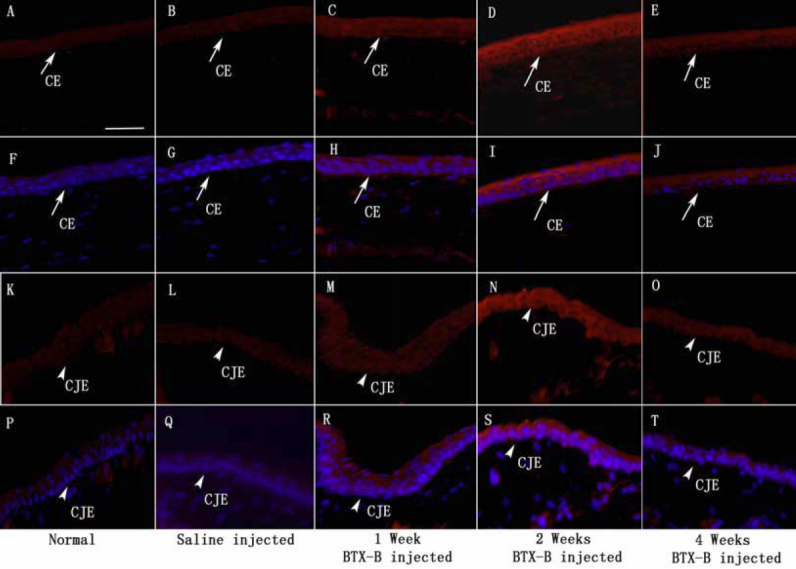
Immunofluorescent staining with IL-1β antibody (red) in corneal epithelia (CE: arrows) and conjunctival epithelia (CJE: arrowheads) during the observation period (**F**-**J** and **P**-**T** with nuclear counterstain in blue). Increased staining intensity for IL-1β in CE and CJE was shown in BTX-B injected mice (1, 2, and 4 weeks post-injection), with most intense labeling at 2 weeks post-injection. Very weak staining in CE and CJE was detected in normal and saline-injected mice. Negative and isotype controls, which did not stain, were omitted. Scale bar: 50 µm.

**Figure 4:**
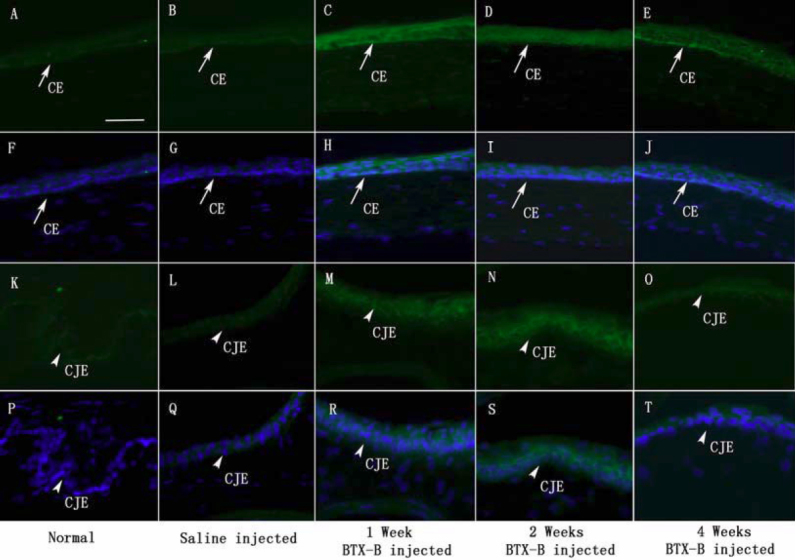
Immunofluorescent staining with TNF-α specific antibody (green) in corneal epithelia (CE: arrows) and conjunctival epithelia (CJE: arrowheads) during the observation period. Nuclear counterstain in blue. Increased staining intensity for TNF-α in CE and CJE was detected in BTX-B injected mice (1, 2, and 4 weeks post-injection). Very weak staining in CE and CJE was observed in normal and saline-injected mice during the observation period (**F**-**J** and **P**-**T** with nuclear counterstain in blue). Images of isotype and negative controls were omitted (non-staining). Scale bar: 50 µm.

**Figure 5:**
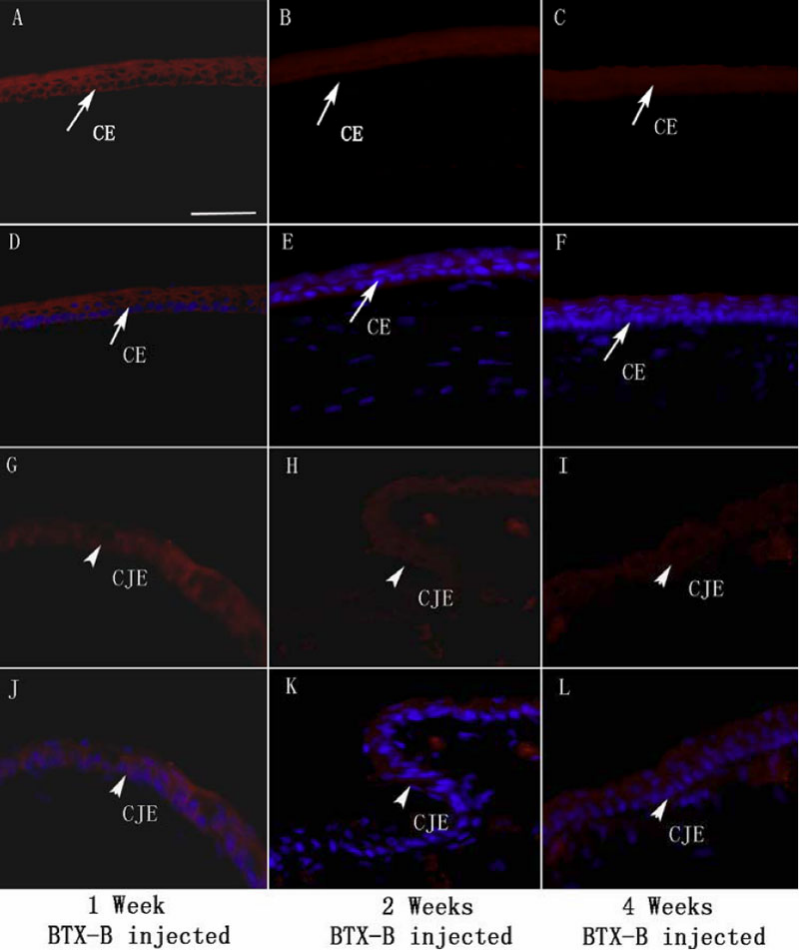
Immunofluorescent staining with IL-1β antibody (red) in corneal epithelia (CE: arrows) and conjunctival epithelia (CJE: arrowheads) were detected in FML-treated mice at 1 week BTX-B post-injection, decreased significantly at 2 and 4 weeks post-injection compared to other groups (**D**-**F** and **J**-**L** with nuclear counterstain in blue). Negative and isotype controls, which did not stain, were not shown. Scale bar: 50 µm.

**Figure 6:**
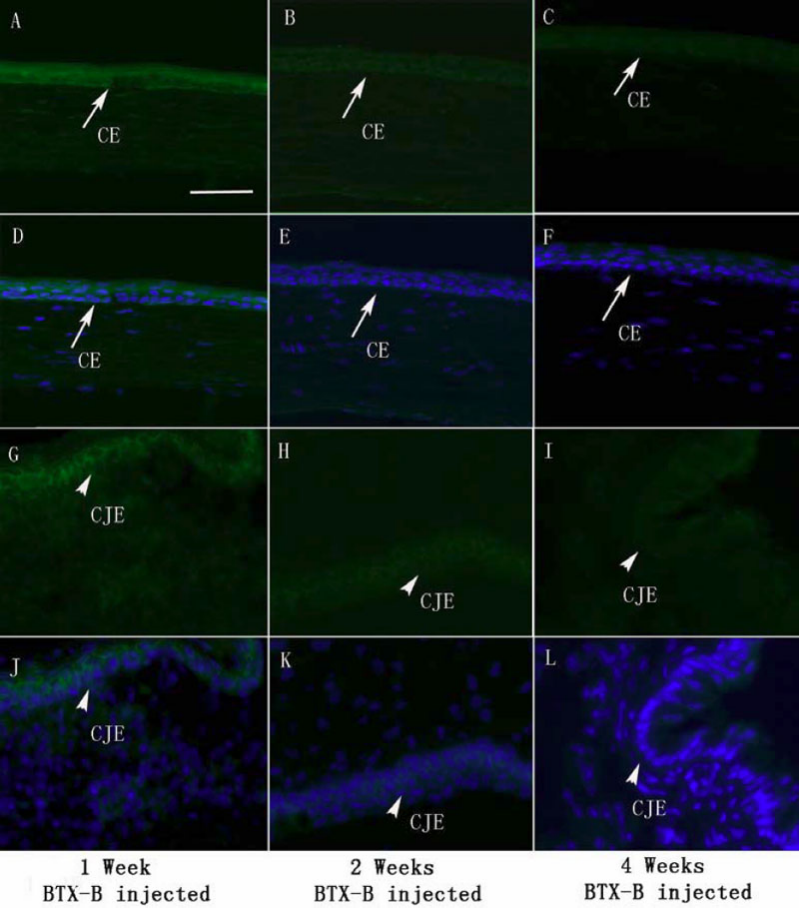
Immunofluorescent staining with TNF-α specific antibody (red) in corneal epithelia (CE: arrows) and conjunctival epithelia (CJE: arrowheads) were detected in FML-treated mice at 1 week BTX-B post-injection, and decreased significantly at 2 and 4 weeks post-injection compared to other groups (**D**-**F** and **J**-**L** with nuclear counterstain in blue). Images of isotype and negative control were omitted (no staining). Scale bar: 50 µm.

**Figure 7:**
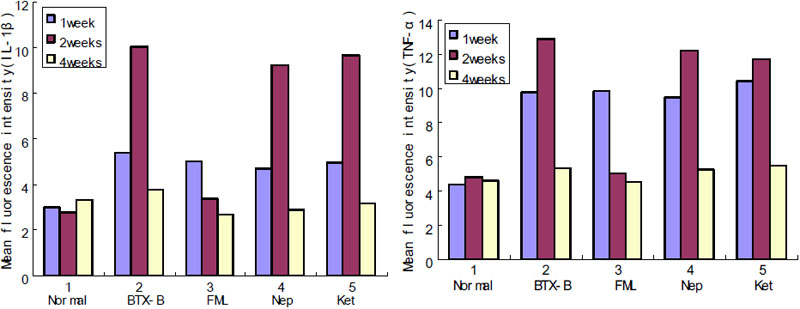
Quantification of fluorescence intensity for IL-1β and TNF-α. Mean fluorescence intensity for IL-1β and TNF-α in different groups was quantified and compared among them. BTX-B-injected mouse cornea and conjunctiva showed IL-1β and TNF-α staining significantly higher than that of normal and saline-injected mice at the 1 and 2 week time points (p<0.01, *t*-test). FML-treated mice with BTX-B injection revealed a 66% and 60% reduction in IL-1β and TNF-α staining respectively in the corneal and conjunctival epithelia at the 2 week time point (p<0.01, *t*-test). FML- fluorometholone-treated group, Nep- nepafenac-treated group, Ket- ketorolac-treated group.

We performed immunolabeling of normal, saline-injected and BTX-B-injected mouse cornea and conjunctiva with anti-TNF-α and IL-1β antibodies. Very weak staining in normal control corneal and conjunctival epithelia was detected for IL-1β (Figure 3A,F,K,P) and TNF-α (Figure 4A,F,K,P). Similar staining patterns were observed in saline-injected mice (Figure 3B,G,L,Q, IL-1β staining; Figure 4B,G,L,Q, TNF-α staining), and there was no change during the observation time points in any treatment groups. With quantitative analysis of fluorescence intensity, corneal and conjunctival epithelia of BTX-B-injected mice demonstrated 1.79 fold and 3.32 fold increase in IL-1β staining (Figure 3C-E,H-J,M-O,R-T), 2.24 fold and 2.97 fold increase in TNF-α staining (Figure 4C-E,H-J,M-O,R-T) over normal and saline-injected mice at the 1 and 2 week time points (p<0.01, *t*-test), whereas the staining decreased significantly at the 4-week time point. In topical fluorometholone-treated mice with BTX-B injection, quantification of mean fluorescence intensity revealed a 66% and 60% reduction in IL-1β (Figure 5B,E,H,K, and Figure 7, p<0.01, *t*-test) and TNF-α (Figure 6B,E,H,K, and Figure 7, p<0.01, *t*-test) staining, respectively, in the corneal and conjunctival epithelia at the 2 week time point (after 1 week of fluorometholone treatment), and the staining returned back to baseline at the 4 week time point (Figure 5C,F,I,L and Figure 6C,F,I,L). Topical artificial tears, 0.1% nepafenac, and 0.4% ketorolac did not show obvious effects on TNF-α and IL-1β staining in the corneal and conjunctival epithelia of BTX-B injected mice compared to BTX-B-injected mice without treatment at the 2 and 4 week time points (Figure 7).

## Discussion

We have previously shown increased expression of the pro-inflammatory cytokines TNF-α and IL-1β in ocular surface epithelia of the BTX-B lacrimal gland-injected dry eye mouse model [9]. The current study confirms that topical fluorometholone significantly decreases the protein expression of TNF-α and IL-1β on corneal and conjunctival epithelia in the Botulium toxin B-induced dry eye mouse model, consistent with improved corneal fluorescein staining, but without the significant change in tear production. Topical artificial tears, 0.1% nepafenac, and 0.4% ketorolac did not show significant effects on expression of TNF-α and IL-1β in ocular surface epithelia and had no influence on tear production and corneal staining.

Growing evidence suggests that inflammation plays a key role in the development of dry eye. Evidence includes increased expression of immune activation markers, inflammation cell infiltration and inflammatory cytokines expression in ocular surface epithelia characterized by the increased expression of matrix metalloproteinases (MMPs), TNF-α, and IL-1β in ocular surface epithelia and tear film through activation of the mitogen-activated protein kinase (MAPK) pathways [10-16]. Corticosteroids are strong inhibitors of inflammation and have been proven to decrease the production of IL-1α, IL-1β, and TNF-α by inhibiting MAPK function [5]. In this study, fluorometholone showed significant inhibition of IL-1β and TNF-α expression of ocular surface epithelia and improvement of corneal fluorescein staining in the BTX-B dry eye mouse model, but did not change the tear production. The results are consistent with those of other studies in both humans and animal models [5,17-20].

Topical ophthalmic NSAIDs have become important anti-inflammatory agents and generally been considered safe and effective therapeutic entities. It is well known that NSAIDs produce their clinical effect by inhibiting cyclooxygenase (COX) and thus inhibiting prostaglandin synthesis. However, inhibition of COX is not the only potential mode of action of the NSAIDs [21,22]. As inflammation is a key component in the pathogenesis of dry eye, NSAIDs have recently been evaluated in dry eye clinical trials and animal models. A previous study demonstrated that several topical NSAIDs appeared to improve corneal staining in the BTX-B dry eye mouse model, and another study in an experimental rabbit dry eye model also found that topical ketorolac and nimesulide ha beneficial effects [23,24]. However, in a clinical trial on keratoconjunctivitis with or without Sjogren’s syndrome, topical flurbiprofen had no effects on subjective and objective clinical parameters as well as conjunctival inflammation which is believed to be the primary pathogenesis of dry eye syndrome [17]. These controversial results are not directly comparable because of the different agents and parameters employed. These studies prompted us to investigate NSAIDs in the BTX-B dry eye mouse model. In this current study, we investigated the effect of 0.1% nepafenac, and 0.4% ketorolac on the BTX-B dry eye mouse model and did not find significant beneficial effects on objective parameters like tear production, corneal fluorescein staining and IL-1β and TNF-α expression in ocular surface epithelia although these parameters tended to improve.

The nepafenac, an amide pro-drug, penetrates the cornea and is converted by ocular hydrolases into amfenac, which is the more active metabolite. So nepafenac may have reasonably weak anti-inflammatory action on the ocular surface. There are also other possible mechanisms [22]. Unlike corticosteroids, which exhibit a broader effect on suppression of inflammation, NSAIDs act primarily through the inhibition of the cyclooxygenase enzyme isoforms [21,22]. However, the changes of the cyclooxygenase enzyme system associated with dry eye have not been documented, so benefits from NSAIDs are undetermined [17,22,23]. There has been recent evidence that arachidonic acid pathways may be involved in dry eye disease [25]. The study demonstrated that the activity of the secretory form of phospholipase A2 group 2a (sPLA2-IIa),which is an important enzyme participating in inflammatory reactions including causing TNF-α and IL-1β pre-activation as well as increased PGE2 production, is significantly increased in the tears of dry eye patients [25,26]. However, NSAIDs are not likely to show direct inhibitory effects on sPLA2-IIa. Another possibility for the failure of nepafenac and ketorolac to decrease inflammatory cytokine expression on ocular surface is due to preservative toxicity. In the eye, preservatives in many ophthalmic solutions have been shown to cause ocular cytotoxic effects in vivo and in vitro models [27-31]. These studies demonstrated that preservatives such as benzalkonium chloride (BAK) may induce ocular surface inflammation, allergy, fibrosis, and dry eye syndrome. In the current study, commercial topical NSAIDs containing BAK were used, so the possible anti-inflammatory effect of NSAIDs on ocular surface could be counteracted by preservative toxicity, while strong anti-inflammatory efficacy of fluoromethaolone might overcome this side effect, although fluoromethaolone has preservatives as well. Our previous study detected statistically significant improvement in corneal staining in BTX-B injected nepafenac-treated mice [23]. In the current study, nepafenac appeared to improve the corneal fluorescein staining score but the changes were not significant. The discrepancy might also be caused by factors including the scoring systems of fluorescein staining. Although fluorescein staining is a widely-used diagnostic tool to assess ocular surface epitheliopathy in dry eye, it is primarily a subjective scoring system that is by nature investigator-specific and bias-prone [32,33]. Thus more quantitative and objective measures of corneal staining should be developed in the future.

### Conclusion

In summary, the current study confirms that topical corticosteroid exhibits suppression of inflammatory cytokine expression in ocular surface epithelia in the botulium toxin B-induced murine dry eye model, while NSAIDs appears not to have significant suppressive effects. Further studies using NSAIDs without preservatives and employing more sensitive tests would be helpful to clarify the efficacy of these anti-inflammatory agents in our dry eye model.
